# Efficient Killing of High Risk Neuroblastoma Using Natural Killer Cells Activated by Plasmacytoid Dendritic Cells

**DOI:** 10.1371/journal.pone.0164401

**Published:** 2016-10-07

**Authors:** Martine Cordeau, Assila Belounis, Martin Lelaidier, Paulo Cordeiro, Hervé Sartelet, Sabine Herblot, Michel Duval

**Affiliations:** 1 Groupe de Recherche En Transplantation et Immunologie du Sang de Cordon (GRETISC), Centre de cancérologie Charles-Bruneau, Centre de recherche du CHU Sainte-Justine, Montreal, Canada; 2 Department of Microbiology and Immunology, University of Montreal, Quebec, Canada; 3 Department of Pediatrics, University of Montreal, Quebec, Canada; 4 Department of Biomedical Sciences, University of Montreal, Quebec, Canada; 5 Département d’anatomie et cytologie pathologiques CHU de Grenoble 38043 Grenoble Cedex 9, France; Hospital Infantil Universitario Nino Jesus, SPAIN

## Abstract

High-risk neuroblastoma (NB) remains a major therapeutic challenge despite the recent advent of disialoganglioside (GD2)-antibody treatment combined with interleukin (IL)-2 and granulocyte monocyte-colony stimulating factor (GM-CSF). Indeed, more than one third of the patients still die from this disease. Here, we developed a novel approach to improve the current anti-GD2 immunotherapy based on NK cell stimulation using toll-like receptor (TLR)-activated plasmacytoid dendritic cells (pDCs). We demonstrated that this strategy led to the efficient killing of NB cells. When the expression of GD2 was heterogeneous on NB cells, the combination of pDC-mediated NK-cell activation and anti-GD2 treatment significantly increased the cytotoxicity of NK cells against NB cells. Activation by pDCs led to a unique NK-cell phenotype characterized by increased surface expression of tumor necrosis factor-related apoptosis-inducing ligand (TRAIL), with increased expression of CD69 on CD56^dim^ cytotoxic cells, and strong interferon-γ production. Additionally, NB-cell killing was mediated by the TRAIL death-receptor pathway, as well as by the release of cytolytic granules via the DNAX accessory molecule 1 pathway. NK-cell activation and lytic activity against NB was independent of cell contact, depended upon type I IFN produced by TLR-9-activated pDCs, but was not reproduced by IFN-α stimulation alone. Collectively, these results highlighted the therapeutic potential of activated pDCs for patients with high-risk NB.

## Introduction

Neuroblastoma (NB) is a tumor of the sympathetic nervous system and the most frequent extracranial pediatric solid tumor, occurring mostly in children before 5 years of age [1,2]. Fifty percent of NB patients >1 year of age present a high-risk metastatic disease with poor prognosis. More than one-third of these patients with high-risk NB progress under treatment or relapse despite aggressive therapeutic regimens [3–7], and most of these children will ultimately die from this disease [8–10].

Natural killer (NK) cells play important roles in tumor immunity and tumor immune surveillance [11]. The antitumor functions of NK cells are tightly regulated by the balance of activating and inhibitory signals [12]. The interaction of NK cell-activating receptors such as DNAX accessory molecule 1 (DNAM-1) and natural killer group 2D (NKG2D), with their respective ligands expressed on tumor cells, poliovirus receptor (PVR) and Nectin-2 for DNAM-1, major histocompatibility complex class I-related chain A/B (MICA/B), and UL16-binding proteins (ULBPs) for NKG2D, triggers the release of cytolytic granules by NK cells, leading to tumor cell death. NK cell lytic functions increase following cytokine stimulation or interaction with activated dendritic cells. These stimulations lead to the expression of ligands for death receptors such as Fas ligand (FAS-L) and tumor necrosis factor-related apoptosis-inducing ligand (TRAIL) by NK cells and then to apoptosis through death-receptor pathways. In contrast, NK-cell inhibitory signals are induced by the interaction of inhibitory killer immunoglobulin-like receptors (KIR) or the heterodimer NK group 2A/CD94 expressed by NK cells with human leukocyte antigen (HLA) class I molecules expressed by target cells.

Clinically available immunotherapy for NB is based on the use of monoclonal antibodies against the surface antigen disialoganglioside (GD2) combined with granulocyte/monocyte-colony stimulating factor and interleukin (IL)-2 [7]. The efficacy of the anti-GD2 monoclonal antibodies relies not only upon complement activation, but also on antibody-mediated cellular cytotoxicity (ADCC) mediated by GM-CSF and IL-2-activated NK cells [13–19]. However, this approach has limitations. First, the use of IL-2 is associated with severe side effects. Secondly, IL-2 may not be the best cytokine to activate NK cells in patients as it increases proliferation of T regulatory cells, which could reduce NK-cell antitumor activity [20]. Additionally, NK-cell cytotoxicity is negatively regulated by inhibitory receptors recognizing self-HLA molecules on NB cells [21,22]. These hindrances can explain why one third of the patients relapse after this treatment, underlying the need for alternative approaches to increase NK-cell toxicity against NB.

Tumor relapse and metastasis of NB correlate with the presence of cancer stem cells (CSCs) within the tumor [23,24]. CSCs possess functional characteristics of stem cells such as self-renewal, quiescence, and the ability to spread into multiple metastatic tumors. Therefore, CSCs are usually resistant to chemotherapy that targets actively cycling cells and are responsible for tumor relapse [25–31]. Thus, targeting CSCs is important when designing a novel NK cell-based immunotherapy. Encouragingly, CSCs are sensitive to immunotherapy and, in particular, NK cell-based immunotherapy, due to the expression of NK cell-activating receptor ligands on CSCs [32–35]. Although NB CSC model is complex, CD133 is a surface marker associated with the stem cell-like phenotype [36–40]. In particular, we previously demonstrated that CD133^+^ SK-N-DZ NB cells are enriched in CSCs as assessed by gene expression analysis and orthotopic xenotransplantation in immunodeficient mice [23,41,42] Although not specific of NB CSCs, CD133 is thus currently the best available surface marker associated with NB stem cell-like phenotype.

Plasmacytoid dendritic cells (pDCs) are a specialized dendritic cell subset that orchestrates both innate- and adaptive-immune responses following infection challenge. In response to toll-like receptor (TLR) stimulation by viral RNA or DNA, pDCs produce high concentrations of type I interferons (IFNs), cytokines and chemokines that are strong activators of NK-cell lytic functions [43–46]. Therefore, pDCs are an attractive therapeutic tool to initiate NK cell-mediated antitumor responses.

In this study, we investigated whether TLR-activated pDCs induced NK-cell cytotoxicity against high-risk NB, including CD133^+^ and GD2^−^ cells. We explored the combination of activated pDCs and anti-GD2 antibody and determined the mechanisms of NB lysis by pDC-activated NK cells. Together, our results supported the hypothesis that patients with high-risk NB may benefit from NK-cell stimulation by activated pDCs to increase NK-cell lytic functions against NB cells.

## Materials and Methods

### NB Cell Lines

SJNB-7 cell line was obtained from St. Jude Hospital (Memphis, TN, USA), and SK-N-AS and SK-N-DZ cell lines were purchased from American Type Culture Collection (Manassas, VA, USA). SJNB-7 cells were derived from a primary adrenal gland tumor, while SK-N-AS and SK-N-DZ cell lines were derived from bone-marrow metastases from children with high-risk NB. All cell lines were cultured in Dulbecco's Modified Eagle Medium (Wisent Bioproducts, St-Bruno, Quebec, Canada) supplemented with 10% fetal bovine serum (FBS; Wisent Bioproducts) in a humidified atmosphere (5% CO_2_) at 37°C. Passages were performed when confluence reached 80 to 90%.

### Isolation of Peripheral Blood NK Cells and pDCs

NK cells and pDCs were isolated from peripheral blood samples from healthy adult volunteers. Written informed consent was obtained from all participants in accordance with the Declaration of Helsinki after CHU Sainte Justine's Institutional Review Board approval. After peripheral blood mononuclear cell (PBMC) isolation by gradient centrifugation (Ficoll-Paque Plus, GE Healthcare, Pittsburgh, PA, USA), NK cells and pDCs were purified by negative magnetic selection (StemCell Technologies, Vancouver, BC, Canada) according to manufacturer instructions. The purity of the selected populations was assessed by flow cytometry and was always >95%.

### NK Cell Stimulation

Purified NK cells were cultured in RPMI-1640 (Invitrogen, Burlington, ON, Canada) supplemented with 10% FBS at a concentration of 1 × 10^6^ cells/mL in 96-well round-bottomed plates for 20 h. NK cells were either unstimulated or stimulated by IL-15 (20 ng/mL; Miltenyi Biotec Inc., Auburn, CA, USA), IFN-α (1000 IU/mL; Merck, Kirkland, Quebec, Canada), or IL-2 (20 IU/mL; Novartis Pharmaceuticals Canada, Dorval, Quebec, Canada). For NK/pDC co-cultures, purified NK cells and pDCs were mixed at a ratio of 10:1 in the presence of CpG oligodeoxynucleotides (10 μg/mL; ODN 2216; InvivoGen, San Diego, CA, USA). For trans-well experiments, NK/pDC co-cultures were performed in 24-well plates using ThinCert inserts (Greiner Bio-One GmbH, Frickenhausen, Germany) with a 0.4-μm pore size. For type I IFN-blocking experiments in NK/pDC co-cultures, non-specific binding sites were blocked by adding 2 μg of mouse IgG2a, followed by the addition of mouse anti-human type I IFN-receptor chain 2 and anti-human IFN-α antibodies (20 μg/mL each; PBL Assay Science, Piscataway, NJ, USA).

### NK Cell-Mediated Cytotoxicity Assays

NK cell-mediated cytotoxicity assays were performed by flow cytometry as described previously [47]. Briefly, NB cell lines were labeled with carboxyfluorescein succinimidyl ester (CFSE) or 3,3′-dihexyloxacarbocyanine iodide_(18)_, and 10,000 labeled cells/well were plated in 96-well flat-bottomed plates and incubated overnight to allow cells to adhere. For ADCC experiments, anti-GD2 ch14.18 chimeric monoclonal antibody (mAb; 1 μg/mL; National Cancer Institute, Rockville, MD, USA) was added to NB target cells before the addition of NK cells. For blocking experiments using monoclonal antibodies against TRAIL and DNAM-1, non-specific binding sites on NK cells were blocked using mouse isotype-matched IgG before the addition of anti-human TRAIL and/or DNAM-1 monoclonal antibodies (30 μg/mL; BD Biosciences, Mississauga, ON, Canada). Unstimulated and activated NK cells were incubated in triplicate with target NB cells at different effector:target (E:T) ratios (1:1, 2.5:1, 5:1, and 10:1). After a brief centrifugation to ensure contact between effectors and targets, the plates were incubated at 37°C for 3 h. NB cells were then trypsinized and collected, counting beads and propidium iodide (PI; Invitrogen) were added to each sample, and viable NB cells were counted by flow cytometry using the BD Fortessa system (BD Biosciences, San Jose, CA, USA). SK-N-DZ cells were stained with an anti-CD133-conjugated antibody (Miltenyi Biotec, Auburn, CA, USA). Data analyses were performed using the FlowJo program (Tree Star, Ashland, OR, USA), and the percentages of specific cell lysis were calculated using the following formula:

$$\left[\left(\frac{\begin{array}{c} absolute\;number\;of\;CFSEpos\;PIneg\;cells\;in\;control\;-\\ absolute\;number\;of\;CFSEpos\;PIneg\;cells\;in\;the\;sample \end{array}}{absolute\;number\;of\;CFSEpos\;PIneg\;cells\;in\;the\;control}\right)\right]\times 100\;=\;\%\;of\;specific\;lysis$$

### Phenotypic Analysis of NB Cell Lines and Activated NK Cells

The phenotypes associated with SJNB-7, SK-N-AS and SK-N-DZ cell lines were assessed by flow cytometry using the following conjugated antibodies: HLA-ABC-phycoerythrin (PE) (BD Biosciences, San Jose, CA, USA), TRAIL-R1 (DR4, CD261)-allophycocyanin (APC), TRAIL-R2 (DR5, CD262)-PE (BioLegend, San Diego, CA, USA), CD95 (Fas/Apo-1)-fluorescein isothiocyanate (FITC) (BD Biosciences, San Jose, CA), CD178 (FAS-L, CD95L)-PE (Caltag, Burlington, ON, Canada), CD155-PE (PVR; BioLegend), Nectin-2-PE (CD112; BioLegend), MICA/B-PE (BioLegend), and ULBP-2-PE (R&D Systems, Minneapolis, MN, USA). Dead cells were excluded by 7-AAD) staining (BD Pharmingen, Mississauga, ON, Canada).

Following overnight stimulation, NK cells were labeled with the following antibodies: CD69-FITC, CD95 (Fas)-FITC, CD16-FITC (BD Biosciences, San Jose, CA, USA), CD56-FITC, CD336 (NKp44)-PE (BioLegend), CD253 (TRAIL)-PE, CD178 (FAS-L)-PE, CD314 (NKG2D)-PE, CD16-PE/Cy7 (BD Biosciences, San Jose, CA, USA), CD56-APC, CD335 (NKp46)-Brilliant Violet 421, CD337 (NKp30)-Alexa Fluor 647 (BioLegend), CD96-PE/V770 (Miltenyi), DNAM-1-PE (CD226, BD Biosciences, San Jose, CA, USA), and dead cells were excluded using 7-AAD staining. For perforin and granzyme intracellular staining, NK cells were first stained with the CD56-APC antibody, then fixed and permeabilized with Cytoperm/Cytofix (BD Biosciences, San Jose, CA, USA). Perforin-FITC and granzyme B-PE antibodies (BD Biosciences, San Jose, CA, USA) were then incubated according to manufacturer instructions. All acquisitions were performed on a Fortessa cytometer (BD Biosciences, San Jose, CA, USA), and data analysis was performed using the FlowJo program (Tree Star).

### NK Cell-Degranulation Assay

Degranulation assays were performed as previously described [48]. Briefly, activated NK cells and target cells were mixed at a 1:1 ratio in the presence of the CD107a-PE antibody (BD Biosciences, San Jose, CA, USA) and incubated for 1 h at 37°C. GolgiStop (6 μg/mL; BD Biosciences, San Jose, CA, USA) was then added, and cells were incubated for an additional 4 h at 37°C. When required, NB target cells were first incubated with ch14.18 anti-GD2 mAb (1 μg/mL) before the co-culture with NK cells. After staining with CD56-APC antibody and addition of 7-AAD, surface expression of CD107a was assessed using a Fortessa cytometer (BD Biosciences), and data analysis was performed using the FlowJo program (Tree Star).

### Statistical Analysis

One-way analysis-of-variance tests were used for multiple-group comparisons of paired data, and paired *t* tests were used for single-data comparisons. A *p* < 0.05 (*) was considered significant with a confidence interval of 99% (GraphPad Software, San Diego, CA, USA).

## Results

### NK Cells Stimulated with Activated pDCs Kill Both CD133^+^ and GD2^−^ NB Cells

**Fig 1:**
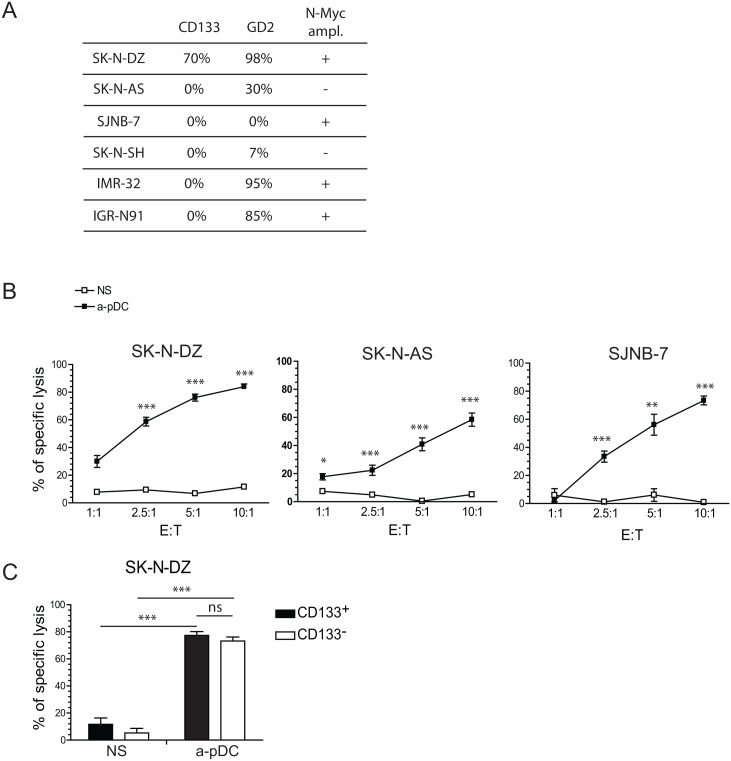
NB resistance to NK cell-mediated killing is overcome by NK-cell stimulation with TLR-9-activated pDCs. A—Phenotypic characteristics of selected NB cell lines. CD133 and GD2 expression were assessed by flow cytometry. B–*In vitro* cytotoxicity assays were performed against SK-N-DZ, SK-N-AS, and SJNB7 NB cell lines. NK cells and pDCs were purified from peripheral blood samples, and NK cells were stimulated with TLR9-stimulated pDCs for 18–24 h prior to the assays. Graphs represent the mean of specific lysis observed in three to five experiments, with error bars representing the standard error of the mean. C—To determine the sensitivity of CSCs to NK cell-mediated killing, SK-N-DZ cells were stained with the anti-CD133 mAb after a 3-h incubation with activated NK cells. Specific lysis of CD133^+^ and CD133^−^ SK-N-DZ cells was determined. Graphs represent the mean of specific lysis observed in three to five experiments, with error bars representing the standard error of the mean. Statistical analyses were performed using one-way analysis of variance. * *p* < 0.05, **p < 0.01, ***p < 0.001. CD, cluster of differentiation; CSC, cancer stem cell; GD2: disialoganglioside; NB, neuroblastoma; NK, natural killer; pDC, plasmacytoid dendritic cell; TLR, toll-like receptor.

NB are heterogeneous tumors with various phenotypes as exemplified by the diversity of GD2 and CD133 expression on NB cell lines derived from patients with high-risk NB (Fig 1A). We selected three NB cell lines (SK-N-DZ, SK-N-AS, and SJNB-7) that differed in their expression of GD2 and CD133 surface markers. We tested the ability of unstimulated or pDC-activated NK cells to kill these NB cell lines using *in vitro* cytotoxicity assays. In the absence of NK-cell activation, all three NB cell lines were resistant to NK cell-mediated lysis. Co-culture of NK cells with TLR-9-activated pDCs significantly increased the killing of NB cell lines, including GD2^−^ SJNB-7 cells (Fig 1B). Within the SK-N-DZ cells, CD133^+^ CSCs exhibited similar sensitivity levels as those observed in CD133^−^ cells to NK cell-mediated lysis when NK cells were stimulated by activated pDCs (Fig 1C). These results demonstrated that, following co-culture with TLR-9 stimulated pDCs, NK cells acquired the capacity to eradicate NB cells, including CSCs and GD2-negative cells.

### The Additive Effect of Activated pDCs and Anti-GD2/IL-2 on NK Cell-Mediated Killing of NB Cells in the Presence of Heterogeneous Expression of GD2

**Fig 2:**
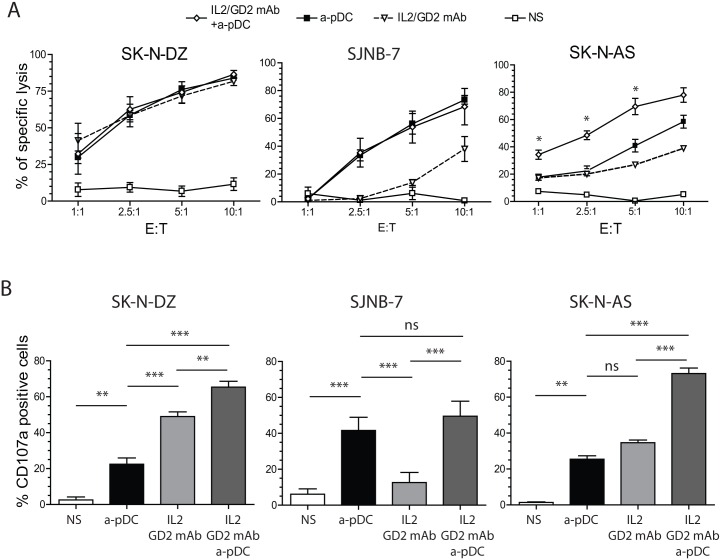
Additive effects of anti-GD2 mAb, ADCC, and pDC-activated NK-cell cytotoxicity against NB cells. A—Cytotoxicity assays were performed against SK-N-DZ, SJNB-7, and SK-N-AS cell lines in the presence or absence of prior incubation of target cells with the anti-GD2 mAb. Effectors were either unstimulated NK cells (NS), NK cells stimulated by overnight incubation with TLR-9-activated pDCs (a-pDC), or NK cells stimulated by low doses of IL-2 alone (IL2/GD2 mAb) or in combination (IL2/GD2 mAb+a-pDC). Graphs present the specific lysis means of three independent experiments, with error bars representing the standard error of the mean. B—NK cell-degranulation assays were performed against SK-N-DZ, SJNB7, and SK-N-AS cells. Unstimulated NK (NS) and stimulated NK cells (a-pDC, IL2/GD2 mAb, or IL2/GD2 mAb+a-pDC) were incubated with target cells at a 1:1 ratio and stained with the anti-CD107a antibody. Graphs represent the means of CD107a MFIs on NK cells (gated on CD56^+^CD3^−^) in three independent experiments. Statistical analyses were performed using one-way analysis of variance. *p < 0.05, **p < 0.01, ***p < 0.001. ADCC, antibody-dependent cellular cytotoxicity; CD, cluster of differentiation; GD2: disialoganglioside; IL, interleukin; mAb, monoclonal antibody; MFI, mean fluorescence intensity; NB, neuroblastoma; NK, natural killer; pDC, plasmacytoid dendritic cell; TLR, toll-like receptor.

Using cytotoxic and degranulation assays, we next compared anti-GD2-targeted ADCC mediated by IL-2-stimulated NK cells with the cytotoxicity levels initiated by pDC-activated NK cells. pDC-activated NK cells and anti-GD2-targeted ADCC were equally efficient at killing GD2-expressing SK-N-DZ cells (Fig 2A). The combination of anti-GD2 mAb with pDC-mediated activation of NK cells did not increase SK-N-DZ lysis, although there was an additive effect of GD2/IL-2- and pDC-activation on granule release against SK-N-DZ cells as measured by CD107a expression on NK cells (Fig 2B). As expected, anti-GD2-mediated ADCC was inefficient at killing GD2-negative SJNB-7 cells, and these cells did not induce granule release by NK cells. Interestingly, these GD2-negative cells were killed by pDC-stimulated NK cells, thereby inducing cytotoxic granule release by pDC-activated NK cells (Fig 2A and 2B).

Finally, we compared anti-GD2-mediated ADCC, pDC activation, and their combination in the killing of SK-N-AS cells, a NB cell line exhibiting heterogeneous expression of GD2 (Fig 2A). The efficacy of anti-GD2-mediated ADCC was much lower than that observed on GD2-positive cells, and SK-N-AS cells were sensitive to NK cells stimulated by activated pDCs. Interestingly, we observed increased killing of SK-N-AS cells by the combination of anti-GD2-mediated ADCC and pDC-mediated NK-cell activation (Fig 2A). This additive effect of ADCC and pDC-mediated NK-cell activation was associated with an increased release of cytotoxic granules (Fig 2B). Therefore, our results demonstrated that activated pDCs and anti-GD2-targeted ADCC exert an additive effect for the killing of a cell line with heterogeneous expression of GD2, leading to NB cell death comparable to that observed in a GD2-positive cell line.

### Activated pDCs Induce CD69 and TRAIL Expression, as well as IFN-γ Production in NK Cells

**Fig 3:**
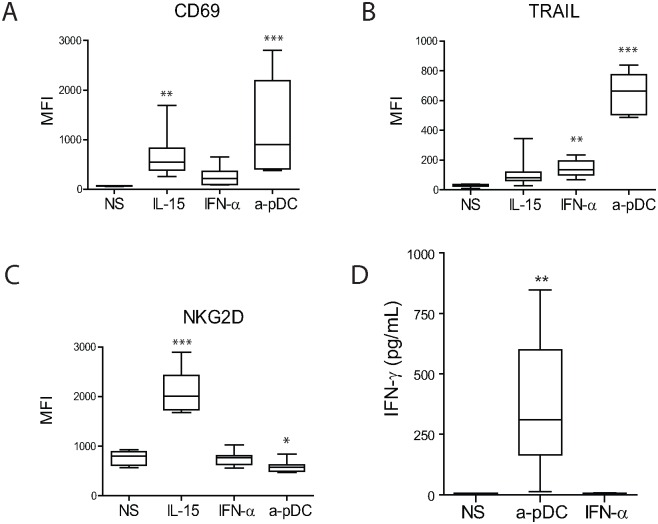
TLR9-stimulated pDCs induce a unique NK-cell phenotype and IFN-γ production. A–C—Phenotype analysis of NK cells following overnight stimulation with IL-15, IFN-α, or co-culture with TLR9-stimulated pDCs. Graphs represent the means of MFIs for (A) CD69, (B) TRAIL, and (C) NKG2D immunostaining, with error bars representing the standard error of the mean from five to eight experiments on NK cells from different donors. D—IFN-γ production was measured by ELISA in the supernatant of NK-cell cultures following overnight stimulation. The means of four to six independent experiments are represented, with error bars representing the standard error of the mean. Statistical analyses were performed using one-way analysis of variance. *p < 0.05, **p < 0.01, ***p < 0.001. CD, cluster of differentiation; ELISA, enzyme-linked immunosorbent assay; IFN, interferon; IL, interleukin; MFI, mean fluorescence intensity; NK, natural killer; NKG2D, natural killer group 2D; pDC, plasmacytoid dendritic cell; TLR, toll-like receptor; TRAIL, tumor necrosis factor-related apoptosis-inducing ligand.

To determine the mechanisms responsible for NK cell-mediated lysis of NB tumors, we assessed the phenotype of NK cells stimulated by either TLR-9-activated pDCs, IFN-α or IL-15. Here, we used IFN-α concentrations (1000 IU/mL) expected to be produced by activated pDCs (4 × 10^4^ cells) in the corresponding experiments [49]. We observed that the upregulation of the CD69-activation marker was significantly enhanced following NK-cell stimulation with TLR-9-activated pDCs as compared to that observed following IFN-α or IL-15 stimulation (Fig 3A). Interestingly, this upregulation of CD69 expression was observed in all CD56^dim^, but not in CD56^bright^ NK cells, whereas CD69 expression was upregulated in only a fraction of CD56^dim^ (30–70% of cells, depending on the donors) and all CD56^brigh^ NK cells upon IFN-α and IL-15 stimulation (S1 Fig).

TLR-9-activated pDCs induced a significantly higher expression of TRAIL on NK cells as compared with that observed following stimulation with IFN-α or IL-15 (Fig 3B). FAS-L was not expressed on pDC-activated NK cells (S2 Fig), and natural cytotoxicity receptors (NKp44, NKp46, or NKp30), NKGD2, granzyme B and perforin were not upregulated (Fig 3C and S2 Fig).

Finally, we observed increased production of IFN-γ by NK cells stimulated by TLR-9-activated pDCs, while IFN-α stimulation did not induce IFN-γ secretion by NK cells (Fig 3D). Collectively, these results indicated that the co-culture of NK cells with TLR-9-activated pDCs induced a unique activated phenotype that cannot be reproduced by IFN-α or IL-15 stimulation alone and is characterized by increased secretion of IFN-γ, high levels of CD69 expression on CD56^dim^ cells, and high levels of surface expression of TRAIL on both CD56^bright^ and CD56^dim^ cells.

### NK-Cell Activation by TLR-9-Activated pDCs Is Independent of Cell Contact

**Fig 4:**
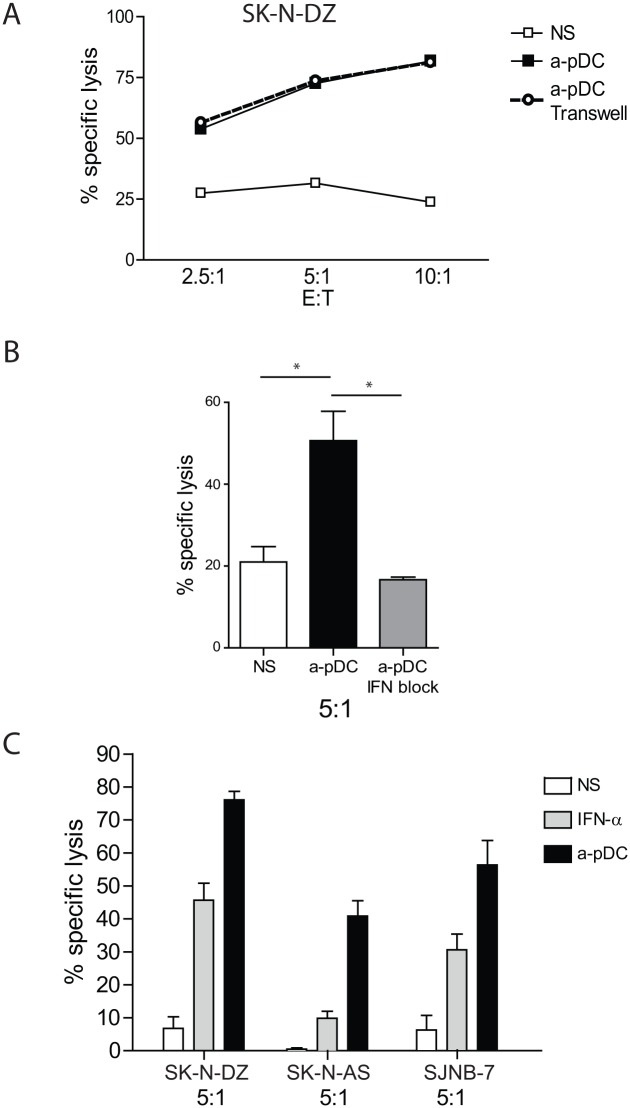
NK-cell activation is independent of contact with pDCs, but depends upon type I IFN production by activated pDCs. A—Analysis of NK-cell cytotoxicity following NK-cell activation with TLR9-stimulated pDCs in contact or in trans-wells. Graphs are representative of three independent experiments. B—Blocking of type I IFN signaling was performed using specific blocking mAbs against IFN-α and type I IFN receptor in NK/pDC co-cultures, followed by cytotoxicity assays performed against SK-N-DZ cells. The graph represents the means of three independent experiments, with error bars representing the standard error of the mean. C—Analysis of NK-cell cytotoxicity following NK-cell activation with TLR9-stimulated pDCs (a-pDC) or IFN-α (1000 IU/mL). The IFN-α concentration was comparable to that estimated under both conditions. Specific lysis of SK-N-DZ, SK-N-AS, and SJNB-7 cells are presented for an E:T ratio of 5:1. Statistical analyses were performed using one-way analysis of variance. *p < 0.05, **p < 0.01, ***p < 0.001. E:T, effector against target; IFN, interferon; IL, interleukin; mAb, monoclonal antibody; NK, natural killer; pDC, plasmacytoid dendritic cell; TLR, toll-like receptor.

We next explored whether soluble factors secreted by activated pDCs or cellular contact between activated pDCs and NK cells was required for NK-cell activation. Levels of NB cell lysis were similar when NK cells and pDCs were cultured in contact or separately in trans-well experiments (Fig 4A). This result indicated that soluble factors secreted by TLR-9-activated pDCs are responsible for NK cell-mediated cytotoxicity against NB.

### NK-Cell Lytic Activity against NB Depends upon IFN-α Production by TLR-9-Activated pDCs, but Is Not Reproduced by IFN-α Stimulation Alone

We then determined the role of type I IFNs in NK-cell stimulation by TLR-9-activated pDCs. We blocked type I IFN signaling using a combination of blocking antibodies against IFN-α and type I IFN receptors. Type I IFN blockade completely abrogated NB cell lysis by pDC-activated NK cells (Fig 4B). To investigate whether NB killing by NK cells was entirely due to IFN-α production by pDCs, we stimulated NK cells with the amount of IFN-α expected to be produced by the 4 × 10^4^ pDCs used in the control experiments. Under these conditions, we observed that NB cell lysis was lower when NK cells were directly activated by IFN-α (Fig 4C). These results indicated that NK-cell lytic activity against NB depends upon type I IFN produced by TLR-9-activated pDCs, but is not reproduced by IFN-α stimulation alone.

### The TRAIL and DNAM-1 Pathways Are Involved in NB Killing by pDC-Activated NK Cells

**Fig 5:**
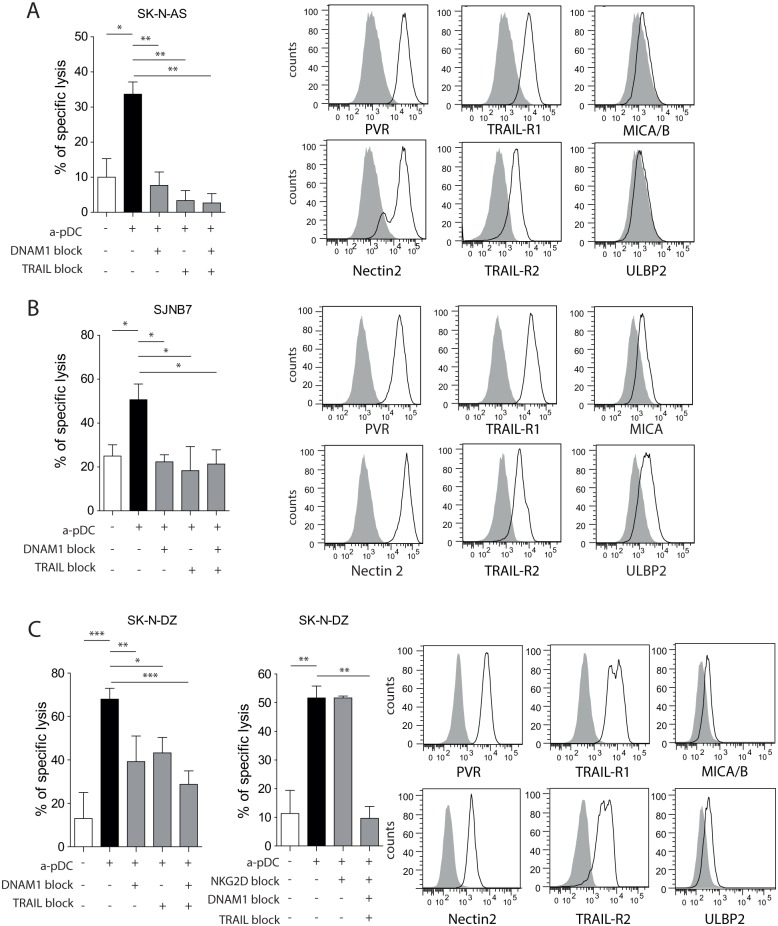
The TRAIL and DNAM-1 pathways are involved in NB killing by pDC-activated NK cells. Cytotoxicity assays against (A) SK-N-AS, (B) SJNB-7, and (C) SK-N-DZ cells were performed in the presence of DNAM-1-, TRAIL-, or NKG2D-blocking antibodies alone or in combination. Bar histograms represent the mean of three independent experiments, with error bars representing the standard error of the mean. The expression of DNAM-1 ligands (PVR and Nectin-2), TRAIL receptors (TRAIL-R1/R2), and NKG2D ligands (MICA/B and ULBP2) was investigated by flow cytometry, and representative histograms are presented for (A) SK-N-AS, (B) SJNB-7, and (C) SK-N-DZ cells. Statistical analyses were performed using one-way analysis of variance. *p < 0.05, **p < 0.01, ***p < 0.001. DNAM-1, DNAX accessory molecule 1; MICA/B, major histocompatibility complex class I-related chain A/B; NB: neuroblastoma; NK, natural killer; NKG2D, natural killer group 2D; pDC, plasmacytoid dendritic cell; PVR, poliovirus receptor; TRAIL, tumor necrosis factor-related apoptosis-inducing ligand; ULBP: UL16-binding protein.

We investigated the role of NK cell-activating receptors, DNAM-1 and NKG2D, as well as death receptor-mediated apoptosis in NK cell-mediated lysis of NB cells using blocking experiments. We observed that all three NB cell lines expressed high levels of DNAM-1 ligands (PVR and Nectin-2) (Fig 5A–5C). Accordingly, blocking the interaction between DNAM-1 and its ligands abrogated NK cell-mediated killing of SK-N-AS and SJNB7 cells (Fig 5A and 5B) and significantly decreased SK-N-DZ cell lysis (Fig 5C).

All three cell lines expressed high levels of both TRAIL receptors, TRAIl-R1 and TRAIL-R2 (Fig 5). We observed that blocking TRAIL interaction with TRAIL-R1 and TRAIL-R2 abrogated NK cell-mediated killing of SK-N-AS and SJNB7 cells and significantly decreased SK-N-DZ cell lysis (Fig 5A–5C). Additionally, NKG2D ligands (MICA/B and ULBP2) were expressed at low levels on SK-N-DZ and SJNB7 cells and were almost absent on SK-N-AS cells. Although NKG2D blockade alone did not decrease SK-N-DZ killing, the complete inhibition of cell lysis required blockage of TRAIL, DNAM-1, and NKG2D. Therefore, our findings suggested that multiple lytic pathways are engaged by pDC-activated NK cells to kill NB tumors, and that the relative role of each pathway depends upon the expression of NK-receptor ligands on target cells.

## Discussion

Our study revealed that TLR-9-activated pDCs induced NK cell-mediated killing of NB cells. We demonstrated that both CD133^+^ CSCs and GD2-negative NB cells are sensitive to pDC-activated NK cell-mediated lysis. We also revealed an additive effect of NK-cell stimulation with activated pDCs and anti-GD2 ADCC for the killing of NB tumors expressing low levels of GD2. NK-cell lytic activity against NB was independent of cell contact, depended upon type I IFN produced by TLR-9-activated pDCs, but was not reproduced by IFN-α stimulation alone. Finally, our results indicated that both cytolytic granule release via NK cell-activating receptors and TRAIL-mediated apoptosis are involved in NB killing by pDC-activated NK cells.

NB may still relapse after multimodal therapy, including aggressive chemotherapy, hematopoietic stem cell transplantation, and anti-GD2 immunotherapy. CSCs are responsible for the long-term maintenance of tumor growth and metastasis due to their self-renewal properties and resistance to chemotherapy [23–31]. Given that immunotherapy is effective on low-cycling cells, CSCs from several cancer types are sensitive to immunotherapy and, in particular to NK cell-based therapy. The sensitivity of CSCs to NK cell-mediated killing is correlated to the expression of stress-induced ligands on CSCs that are recognized by the NKG2D-activator receptor expressed by NK cells [32–35]. Although the concept of CSC has been a matter of debate for NB, CD133 appears to be the best available surface marker associated CSC-like phenotype in NB, CD133^+^ NB cells being enriched in cells with a CSC-like phenotype [36–40]. Here, we showed that CD133^+^ NB cells are efficiently killed by pDC-activated NK cells. Our results are in line with data in other cancers underscoring the role of the activating receptor NKG2D in CSC killing [33,34]. Indeed the killing of the SK-N-DZ cell line (70% CD133^+^) by activated NK cells depended upon NKG2D, while the role of NKG2D was less important to the killing of the CD133^−^ NB cell lines, SK-N-AS and SJNB7.

Our results indicated that the combination of anti-GD2 immunotherapy and NK-cell stimulation with activated pDCs has the potential to eradicate NB tumors exhibiting heterogeneous expression of GD2 and to decrease the potential risk of tumor escape by GD2 downregulation. Indeed, GD2^−^ NB cells were efficiently killed by pDC-activated NK cells, and we observed an additive cytotoxic effect associated with pDC-mediated NK-cell activation and anti-GD2 ADCC against a NB cell line exhibiting heterogeneous expression of GD2. These results suggest that NK cell stimulation with activated pDCs could improve the survival rates of patients with high-risk NB treated with anti-GD2 immunotherapy.

Our results showed that efficient killing of NB cells by activated NK cells was dependent upon both DNAM-1-dependent release of cytotoxic granules and TRAIL-mediated apoptosis. These results confirmed the important role of DNAM-1 in NB killing by NK cells, exemplified by the prognostic value of DNAM-1-ligand expression on NB tumors [50,51]. These findings are in agreement with those reported by Sheard *et al* [44], showing that membrane-bound TRAIL plays an important role in NK cell-mediated cytotoxicity against NB cells and complements the release of cytotoxic granules. While DNAM-1 is constitutively expressed on NK cells, the expression of TRAIL is induced by NK-cell stimulation [52,53]. Importantly, high levels of TRAIL are expressed on NK cells upon stimulation by TLR-activated pDCs. This feature is unique to pDC-mediated NK-cell activation and could not be reproduced by IFN-α or IL-15 stimulation alone. This characteristic allows for the engagement of both the TRAIL-induced apoptosis pathway and DNAM-1-mediated release of cytotoxic granules by activated NK cells against NB cells.

The phenotype of NK cells activated by pDCs is characterized by two other features. First, the expression of the activation marker CD69 by CD56^dim^ cells signals activation of this cytolytic subset [54]. Secondly, pDC-activated NK cells secrete high levels of IFN-γ that synergize with TRAIL/death-receptor pathways. Indeed, IFN-γ promotes TRAIL-mediated cleavage of caspase-8 in NB cell lines [55,56]. Furthermore, IFN-γ can serve as a link to adaptive immunity, given that 5 days of systemic IFN-γ therapy increased T cell trafficking into tumor-biopsy specimens from patients with high-risk NB [57].

Additionally, we showed that the activation of NK cells by pDCs was dependent upon soluble factors secreted by pDCs. From a therapeutic viewpoint, it is, therefore, important to determine whether the effect of activated pDCs on NK cells can be reproduced by a single cytokine, as its administration would be simpler and more affordable than adoptive cell therapy. Because pDCs are specialized cells that produce large amounts of type I IFN in response to stimulation, the best candidate among pDC-secreted cytokines is IFN-α [58]. Accordingly, we found that type I IFN blockade completely abrogated NK-cell activation and cytotoxicity against NB, indicating that type I IFNs are required for pDC-induced NK-cell activation. However, NK-cell activation and cytotoxicity against NB were not reproduced by IFN-α stimulation alone, even at a dosage corresponding to that secreted by the number of pDCs used in these experiments. Therefore, we hypothesized that pDC-induced NK-cell stimulation is due to a combination of type I IFNs or that several other pro-inflammatory cytokines are involved. The dependency on type I IFNs may also be explained by the blockade of the IFN-α autocrine positive feedback loop needed for pDC activation [59]. Thus, it is unlikely that the administration of a single cytokine could reproduce the effect of activated pDCs on NK cell-mediated NB killing. Experiments are currently underway to test this hypothesis.

Our results contrast with those published by Perez-Martinez *et al*. showing that blood dendritic cells, including pDCs, suppressed NK cell functions and increased the risk of leukemia relapse after hematopoietic stem cell transplantation [60]. This discrepancy could be explained by several experimental differences between our study and that of Perez-Martinez *et al*. Indeed, we used the physiological NK:pDC ratio of 10:1 in our experiments, while a ratio of 2:1 was used in Perez-Martinez’ study. Moreover, we used negative selections for both pDC and NK cell purification to avoid alteration of their properties. Since our results are in good agreement with the pDC-induced NK cell activation previously described [43], we are confident that NK-cell stimulation by activated pDCs constitutes a unique therapeutic strategy to fight NB cells.

pDC activation can be induced *in vivo* by the administration of clinical-grade TLR-ligands [61]. However, the efficacy of this approach depends upon the number and function of pDCs in patients. We showed that, following cord-blood or allogeneic bone-marrow transplantation, pDC numbers or functions are altered for several months [49]. Whether aggressive chemotherapy, followed by autologous hematopoietic stem cell transplantation, affects pDC function and numbers in patients with high-risk NB remains to be determined. Alternatively, activated pDCs may be adoptively transferred to induce NK-cell responses. We recently developed a unique method of human pDC expansion from CD34^+^ hematopoietic stem cell progenitors [46,49]. These *in vitro* differentiated pDCs are as efficient as peripheral blood pDCs at inducing NK-cell antitumor activity *in vitro*. We further demonstrated that adoptive transfers of *in vitro* differentiated and activated pDCs control the development of acute lymphoblastic leukemia in humanized mice, proving the feasibility of this therapeutic approach [46]. Experiments are underway to determine whether similar therapeutic efficacy can be obtained against NB.

Our novel approach of NK-cell stimulation by activated pDCs led to the efficient killing of both CSCs and GD2^−^ NB cells and increased the efficacy of anti-GD2-mediated ADCC for NB tumors exhibiting heterogeneous expression of GD2. These results pave the way for novel immunotherapeutic strategies combining anti-GD2 mAb with pDC-mediated activation of NK cells in patients with high-risk NB to reduce the risk of relapse.

## Supporting Information

S1 Fig Flow cytometry analysis of NK cell phenotype following overnight stimulation. Representative dot plot analysis of immunostaining with anti-CD56, anti-CD69, anti-TRAIL antibodies (gated on CD3^-^ cells).
(EPS)

S2 Fig NK cell phenotype was analyzed by flow cytometry following overnight stimulation with IL-15, IFN-α or activated pDCs. Box plots represent the distribution of the median fluorescence intensity (MFI) of the indicated markers (NKp30, NKp46, NKp44, Fas-L, Perforin and Granzyme B). Statistical analyses were performed using one-way analysis of variance. * p < 0.05, **p < 0.01, ***p < 0.001.
(EPS)

## Abbreviations

ADCC: antibody-dependent cellular cytotoxicity
CD: cluster of differentiation
CFSE: carboxyfluorescein succinimidyl ester
CSC: cancer stem cell
DNAM-1: DNAX accessory molecule 1
E:T: effector against target
FAS-L: Fas ligand
FBS: fetal bovine serum
GD2: disialoganglioside
HLA: human leukocyte antigen
IFN: interferon
IL: interleukin
KIR: killer immunoglobulin-like receptor
MICA/B: major histocompatibility complex class I-related chain A/B
NB: neuroblastoma
NK: natural killer
NKG2D: natural killer group 2D
PBMC: peripheral blood mononuclear cell
pDC: plasmacytoid dendritic cell
PE: phycoerythrin
PI: propidium iodide
PVR: poliovirus receptor
TLR: toll-like receptor
TRAIL: tumor necrosis factor-related apoptosis-inducing ligand
ULBP: UL16-binding protein
